# Multi-Omics and Molecular Docking Reveal That Oats and Oat Bran Alleviate Chronic Colitis Via IL-17 Pathway Modulation

**DOI:** 10.3390/nu18030407

**Published:** 2026-01-26

**Authors:** Wen Duan, Tong Li, Yuyu Zhang, Baoguo Sun, Rui Hai Liu

**Affiliations:** 1 Key Laboratory of Geriatric Nutrition and Health, Beijing Technology and Business University, Ministry of Education, Beijing 100048, China; 15754367187@163.com (W.D.); zhangyuyu@btbu.edu.cn (Y.Z.); 2 Key Laboratory of Flavor Science of China General Chamber of Commerce, Beijing Technology and Business University, Beijing 100048, China; 3 Department of Food Science, Cornell University, Ithaca, NY 14853, USA; tl24@cornell.edu

**Keywords:** oats, IBD, metabolite, molecular docking, IL-17 pathway

## Abstract

Background/Objectives: Diet plays a critical role in the development of inflammatory bowel disease (IBD). Our previous work demonstrated that oats and oat bran alleviate dextran sulfate sodium (DSS)-induced colitis in mice by modulating the gut microbiota. Methods: To further explore the underlying mechanisms, this study combined metabolomic and transcriptomic analyses to systematically compare the effects of whole oats and oat bran interventions on chronic colitis. Results: Untargeted metabolomics analysis identified three key metabolites, ursodeoxycholic acid, 3-(3-hydroxyphenyl)propionic acid, and avenanthramide C. The interactions between these metabolites and core proteins of the IL-17 signaling pathway (IL-17A, TRAF6, and ACT1) were evaluated via molecular docking. Transcriptomic and RT-qPCR analyses revealed that both oats and oat bran interventions modulated the IL-17, PI3K-Akt, and TNF signaling pathways. These treatments significantly upregulated the expression of tight junction proteins (claudin-1, claudin-5, occludin) while reducing levels of inflammatory cytokines and chemokines. Molecular docking results demonstrated stable binding between the three metabolites and target proteins primarily through hydrogen bonding and electrostatic interactions, with ursodeoxycholic acid exhibiting the highest binding affinity. Conclusions: Collectively, these findings suggest that oats and oat bran may alleviate chronic colitis by modulating the IL-17 signaling pathway and enhancing intestinal barrier function.

## 1. Introduction

Inflammatory bowel disease (IBD) comprises a group of chronic, nonspecific inflammatory conditions of the gastrointestinal tract, characterized by recurrent episodes of intestinal inflammation and mucosal healing. These pathological processes lead to debilitating symptoms including weight loss, abdominal pain, diarrhea, and hematochezia [1]. The dextran sulfate sodium (DSS) induced murine model of colitis is extensively utilized in IBD research due to its ability to replicate key histopathological features of human ulcerative colitis (UC), such as epithelial barrier disruption, immune system activation, and inflammatory cell infiltration [2]. DSS exerts direct cytotoxic effects on intestinal epithelial cells and lymphocytes, thereby compromising mucosal integrity and triggering a robust immune response. This response is characterized by the release of pro-inflammatory cytokines and chemokines, recruitment of neutrophils and macrophages, and progression to chronic colitis [2].

Current treatments for IBD frequently come with significant side effects, including hepatorenal toxicity, drug resistance, and hypersensitivity reactions [3]. Consequently, there is increasing interest in the development of alternative or adjunctive therapeutic strategies that are both safe and accessible, while also improving patients’ quality of life and effectively managing clinical symptoms [3]. In this context, dietary interventions and functional foods have gained attention as promising therapeutic options. Epidemiological studies consistently demonstrate that a high intake of dietary fiber, particularly from whole grains is associated with a reduced risk of chronic inflammatory conditions, including cardiovascular disease, type 2 diabetes, and colorectal cancer [4]. Conversely, Western diets characterized by high consumption of refined grains, processed foods, and added sugars, alongside a low intake of vegetables and whole grains-are believed to contribute to systemic inflammation [5]. Thus, nutritional modulation represents a practical and evidence-based approach to mitigating inflammatory responses and supporting disease management in patients with IBD.

Whole grains, such as oats, are rich in bioactive compounds, including phytochemicals, dietary fiber, vitamins, and minerals-that contribute to a wide range of health benefits. These include improved digestive function, reduced systemic inflammation, enhanced immune responses, and a lowered risk of chronic diseases [6]. Oats are particularly notable for their high content of β-glucan, a soluble dietary fiber, as well as polyphenols and other bioactive constituents [7]. Emerging evidence suggests that oat derived β-glucan and polyphenols play critical roles in modulating glycemic responses and shaping gut microbiota composition [8]. Clinical studies have shown that oat bran supplementation (60 g/day) increases fecal butyrate concentrations and alleviates symptoms such as abdominal pain and acid reflux in patients with ulcerative colitis (UC) in remission, supporting its potential role in microecological precision nutrition [9]. Preclinical studies further indicate that oat β-glucan fractions attenuate colitis by reducing leukocyte infiltration, lowering malondialdehyde (MDA) levels, promoting nuclear translocation of Nrf2, and inhibiting activation of the TLR4/NF-κB signaling pathway [10]. Notably, whole oats which contain a complex matrix of vitamins, flavonoids, phenolic acids, and polysaccharides may exert more potent anti-inflammatory effects than isolated components alone. Although oats and oat bran are derived from the same grain, their bioactive compounds are distributed differently. Oat bran, which is derived from the bran layer, is rich in dietary fiber, particularly β-glucan, and polyphenols. In contrast, whole oats contain the endosperm, bran, and germ, thus preserving a broader range of nutrients [11].

The gut microbiota and its metabolic products are essential for maintaining intestinal homeostasis. In patients with IBD, microbial diversity is markedly reduced, and metabolite profiles are significantly altered [12,13]. Microbiota-derived metabolites such as short chain fatty acids (SCFAs), bile acids, and tryptophan derivatives-are essential for immune regulation and modulation of inflammatory responses, often acting through interactions with pattern recognition receptors [14,15]. Studies have shown that during colitis, there is a decrease in tryptophan and its derivatives, while bile acid levels are elevated [16]. Transcriptomic approaches such as RNA sequencing (RNA-Seq) have provided insights into the molecular mechanisms by which bioactive compounds modulate signaling pathways and gene expression networks to alleviate colitis [17]. Li et al. (2025) [18] demonstrated that Phyllanthus emblica pectin polysaccharide (PEP-1) enhances intestinal barrier function by reducing serum levels of xanthine and sphingosine, downregulating NF-κB signaling and pro-inflammatory cytokines, and upregulating the expression of tight junction proteins. Transcriptomic analysis further revealed that PEP-1 exerts synergistic anti-inflammatory and barrier-protective effects by modulating multiple pathways, including sphingolipid metabolism, purine metabolism, and spermine biosynthesis [18]. Collectively, these findings underscore that targeted modulation of gut microbiota composition and metabolism represents a promising strategy for mitigating colonic inflammation. However, the underlying mechanisms remain incompletely understood and warrant further investigation through integrated multi-omics analyses. Our preliminary studies have demonstrated that oats and oat bran can help alleviate DSS induced ulcerative colitis in mice, possibly by modulating the gut microbiota [19].

To determine whether the anti-inflammatory effects of oats and oat bran are mediated by microbiota derived metabolites and specific signaling pathways, we employed an integrated multi-omics approach. By combining metabolomic and transcriptomic analyses, we identified key metabolites and signaling pathways that were significantly altered in DSS induced chronic colitis in mice after oats and oat bran intervention. We further validated the expression of genes and proteins related to these pathways using quantitative real-time PCR (RT-qPCR) and immunohistochemistry (IHC). Additionally, molecular docking analysis was conducted to explore how the identified metabolites interact with their potential protein targets, providing insights into the protective mechanisms of oats and oat bran against chronic colonic inflammation. This study aims to systematically assess the distinct effects of whole oats and oat bran on colitis progression and to uncover the molecular mechanisms involved through metabolomic and transcriptomic profiling.

## 2. Materials and Methods

### 2.1. Materials and Reagents

Oats and oat bran were supplied by Inner Mongolia Yangufang Ecological Agricultural Science and Technology (Group) Co., Ltd. (Shanghai, China). Dextran sulfate sodium (DSS; molecular weight 40,000 Da) was obtained from MP Biomedicals, Inc. (Irvine, CA, USA). The NEXTFLEX^®^ Rapid DNA Extraction Kit was purchased from Bioo Scientific in the United States (Austin, TX). DPEC water was purchased from Bioengineer Bioengineering (Zurich, Switzerland). The 5× protein reducing buffer and SDS-PAGE gel preparation kit were procured from Sevier Co. PrimeScript™ (Wuhan, China) RT kits were obtained from Takara Biotechnology (Dalian) Co., Ltd. (Dalian, China). Primary antibodies against NF-κB, TRAF6, IL-17 were purchased from Affinity Biosciences Ltd. (Changzhou, China).

### 2.2. Animals and Experimental Design

Animal care and handling procedures were conducted in accordance with established protocols reported in the literature [19]. A murine model of colitis was induced using previously described methods. All experimental procedures were approved by the Ethics Review Board of South China Agricultural University (Permit No. SCAU-2022B201) and adhered to the Guide for the Care and Use of Laboratory Animals issued by the National Research Council. The study also complied with the ARRIVE (Animal Research: Reporting of In Vivo Experiments) guidelines to ensure transparency and reproducibility. A total of 80 male C57BL/6J mice were randomly assigned to eight groups (*n* = 10 per group): a blank control group, a model control group, three oat intervention groups (low, medium, and high dose: OATL, OATM, OATH), and three oat bran intervention groups (low, medium, and high dose: FIBL, FIBM, FIBH). The administration parameters for DSS, as well as the dosage details for oats and oat bran, are outlined in a previously published article [19]. The control groups were fed a standard AIN-93G diet, while the experimental groups received modified diets supplemented with specific proportions of oat or oat bran. Biochemical data for these animals have been reported in our previous study [19].

### 2.3. Colonic Mucus Layer Examination

Colonic tissue sections were subjected to PAS staining to assess mucus layer integrity. Sections were first incubated with periodic acid solution for 10 min to oxidize carbohydrate components. Following a thorough rinse with distilled water, Schiff’s reagent was applied for 10 min to visualize mucopolysaccharides. The slides were then counterstained with hematoxylin for 1 min to highlight nuclei, followed by rinsing under running water. After dehydration through graded alcohols and clearing in xylene, the sections were sealed with coverslips. Histological evaluation and image acquisition were performed using a light microscope.

### 2.4. Untargeted Metabolomic Profiling of Fecal Samples

Untargeted metabolomic analysis of fecal samples was performed following established protocols as previously described [20]. Approximately 25 mg of fecal material was accurately weighed, to which 100 μL of purified water and 2–3 grinding beads were added. The mixture was homogenized under low-temperature conditions until a uniform suspension was achieved. Subsequently, 400 μL of methanol/acetonitrile (1:1, *v*/*v*) and an additional set of 2–3 grinding beads were added. The sample was vortexed for 30 s, repeated three times, and incubated at −40 °C for 1 h. Following incubation, samples were centrifuged at 13,000× *g* for 15 min at 4 °C, and the supernatant was carefully collected for further analysis. Equal aliquots from each sample were combined to generate a quality control (QC) sample. Metabolite separation was conducted using a Waters ACQUITY UPLC BEH Amide column (2.1 mm × 50 mm, 1.7 μm particle size) (Waters, Milford, MA, USA). MS data were acquired on an Orbitrap Exploris 120 mass spectrometer (Thermo Scientific, Waltham, MA, USA). Detailed mass spectrometry parameters were adopted from Li et al. [18]. Raw data files were converted to the mzXML format using ProteoWizard and subsequently processed using the XCMS package (v3.2) in R for peak detection, alignment, and quantification. Metabolite identification was performed by matching MS/MS fragmentation spectra against an in-house secondary mass spectrometry database (BiotreeDB, v2.1). Multivariate statistical analyses, including principal component analysis (PCA) and partial least squares discriminant analysis (PLS-DA), were conducted. Kyoto Encyclopedia of Genes and Genomes (KEGG) pathway enrichment analysis was also performed to identify relevant metabolic pathways. Metabolites with a *p*-value < 0.05 and fold change > 2.0 were considered statistically significant.

### 2.5. Transcriptomic Analysis of the Colon Tissue

Colon tissue samples from each experimental group were collected, immediately snap-frozen in liquid nitrogen, and transported to Shanghai Meiji Biomedical Technology Co., Ltd. (Shanghai, China) for transcriptomic analysis. Total RNA was extracted from the colon tissues, and its concentration and purity were evaluated using a NanoDrop 2000 spectrophotometer (Thermo Scientific, Waltham, MA, USA). RNA integrity was assessed through agarose gel electrophoresis. High-throughput sequencing of RNA fragments was performed on the Illumina NovaSeq 6000 platform (Illumina Inc., San Diego, CA, USA).

Raw sequencing reads underwent quality filtering and trimming using fastp software (0.19.5) to remove low-quality bases and adapters. Subsequent analyses included gene expression quantification, structural characterization, and identification of novel transcripts. Non-redundant unigenes were annotated by aligning sequences to reference databases using BLAST+ (Version 2.9.0). Differential gene expression analysis was performed using the DESeq2 package, with differentially expressed genes (DEGs) defined by a threshold of |log_2_ fold change| ≥ 1 and adjusted *p*-value < 0.05. Functional enrichment of DEGs was assessed using Kyoto Encyclopedia of Genes and Genomes (KEGG) pathway analysis. Visualization and network analysis of enriched pathways were carried out using the ClueGO and CluePedia plugins in Cytoscape 3.7.1.

### 2.6. RT-qPCR for Gene Expression Validation

To validate the RNA-Seq results, twelve differentially expressed genes were selected for quantitative real-time PCR (RT-qPCR) analysis. Colonic tissue samples were accurately weighed and thoroughly homogenized in lysis buffer, followed by total RNA extraction using standard protocols. The extracted RNA was stored at −80 °C until further analysis. RNA concentration and purity were evaluated using a NanoDrop 2000C spectrophotometer. Complementary DNA (cDNA) was synthesized from the isolated RNA using the PrimeScript™ RT kit (Takara Biotechnology, Dalian, China), according to the manufacturer’s instructions. Quantitative analysis of gene expression was performed using RT-qPCR, and relative expression levels were calculated using the 2^−ΔΔCt^ method, with Gapdh used as the reference housekeeping gene. Primer sequences used for target gene amplification are listed in Table 1.

### 2.7. Immunohistochemistry (IHC) Assay

Colonic tissue samples were fixed, dehydrated, embedded in paraffin, and sectioned. After deparaffinization, antigen retrieval was performed using EDTA buffer (pH 8.0). The sections were then rinsed three times with phosphate-buffered saline (PBS) for 5 min each and subsequently blocked with bovine serum albumin (BSA) for 30 min at room temperature to prevent nonspecific binding. Following the blocking step, tissue sections were incubated overnight at 4 °C with primary antibodies targeting TRAF6, IL-17, and NF-κB. The next day, after another set of PBS washes (3 × 5 min), the sections were incubated with a fluorophore-conjugated secondary antibody for 50 min in the dark. Afterward, the sections were counterstained with DAPI for 10 min in the dark to visualize cell nuclei, followed by three additional PBS washes. To minimize background fluorescence, an autofluorescence quencher was applied, and sections were rinsed under running water for 5 and 10 min, respectively. Finally, the slides were sealed and examined under a fluorescence microscope for image acquisition and analysis.

### 2.8. Molecular Docking Analysis of Key Oat and Bran-Derived Metabolites Targeting Core Proteins

Based on the integrated metabolomics profiling, three metabolites-ursodeoxycholic acid, 3-(3-hydroxyphenyl)propionic acid, and avenanthramide C were selected for molecular docking analysis. Molecular docking was performed using AutoDock 4.2.6 to systematically evaluate the binding affinities and interaction mechanisms between ligands and target proteins. Amino acid sequences for IL-17RA, Act1, and TRAF6 were retrieved from the NCBI database, and homology models of their three-dimensional (3D) structures were constructed using the SWISS-MODEL online platform. The 3D conformations of the selected ligands were obtained from the PubChem database. Docking simulations were performed by calculating binding free energies to assess the strength and stability of ligand–protein interactions, as well as to identify key binding pockets and interaction residues. The 3D structure of human IL-17RA protein (PDB ID: 4NUX) was obtained from the Protein Data Bank (https://www.rcsb.org/). Prior to docking, all crystallographic water molecules were removed from protein structures (PDB format), and hydrogen atoms were added using Reduce (http://kinemage.biochem.duke.edu/software/reduce, accessed on 7 December 2025). Proteins were treated as rigid structures, whereas ligand molecules were given full torsional flexibility. Docking input files were prepared using AutoDock Tools 1.5.6, including the assignment of Kollman charges to proteins and Gasteiger charges to ligands. As the exact ligand-binding sites were not predefined, an exploratory grid box covering the entire protein surface was constructed with a grid spacing of 1.0 Å. Preliminary docking suggested that ligand binding predominantly occurred near the β-domain region of the proteins. To refine the analysis, a focused grid box was centered over this region, defined as 20 × 20 × 20 grid points in the x, y, and z dimensions, respectively, with the same grid spacing. The docking pose with the lowest binding free energy was selected as the optimal conformation. Resulting ligand–protein complexes were visualized and analyzed using PyMOL (v2.5) and Discovery Studio 2019, which enabled identification of hydrogen bonds, hydrophobic interactions, and electrostatic forces contributing to binding stability [21].

### 2.9. Statistical Analysis

One-way analysis of variance (ANOVA) was conducted to assess significant differences between the control and treatment groups. A *p* ˂ 0.05 was considered statistically significant. Quantification of immunohistochemical staining was performed using ImageJ software (v1.54). Transcriptomic data were analyzed using the Meggie BioCloud platform (www.majorbio.com).

## 3. Results

### 3.1. Oat and Oat Bran Ameliorate Colonic Mucus Layer Damage

**Figure 1:**
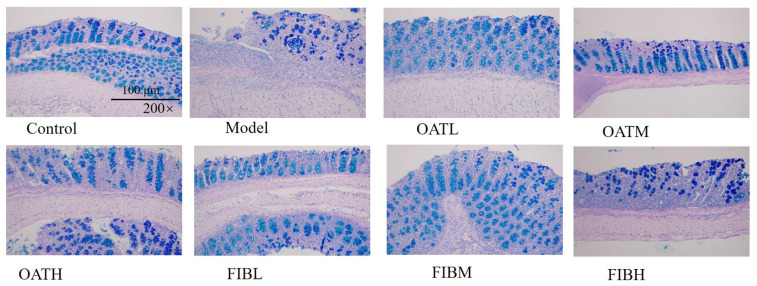
Effects of oat and oat bran on the colon tissue in mice with images of PAS staining.

Previous studies have shown that colitis is associated with a marked reduction in goblet cell numbers, leading to significantly decreased mucus secretion and consequent thinning or partial loss of the protective mucus layer. In Figure 1, the model group exhibited a substantial decline in goblet cell numbers, reduced mucus production, disruption of the mucus layer architecture, and severe damage to the intestinal mucosal barrier, compared to the control group. These findings indicate that DSS treatment induces considerable impairment of the intestinal mucosal barrier in mice. In contrast, mice treated with various doses of oats and oat bran showed a significant restoration of goblet cell populations, enhanced mucus secretion, and improved integrity of the colonic mucosal barrier, compared to the model group.

### 3.2. Effects of Oats and Oat Bran on Fecal Metabolite Profile

**Figure 2:**
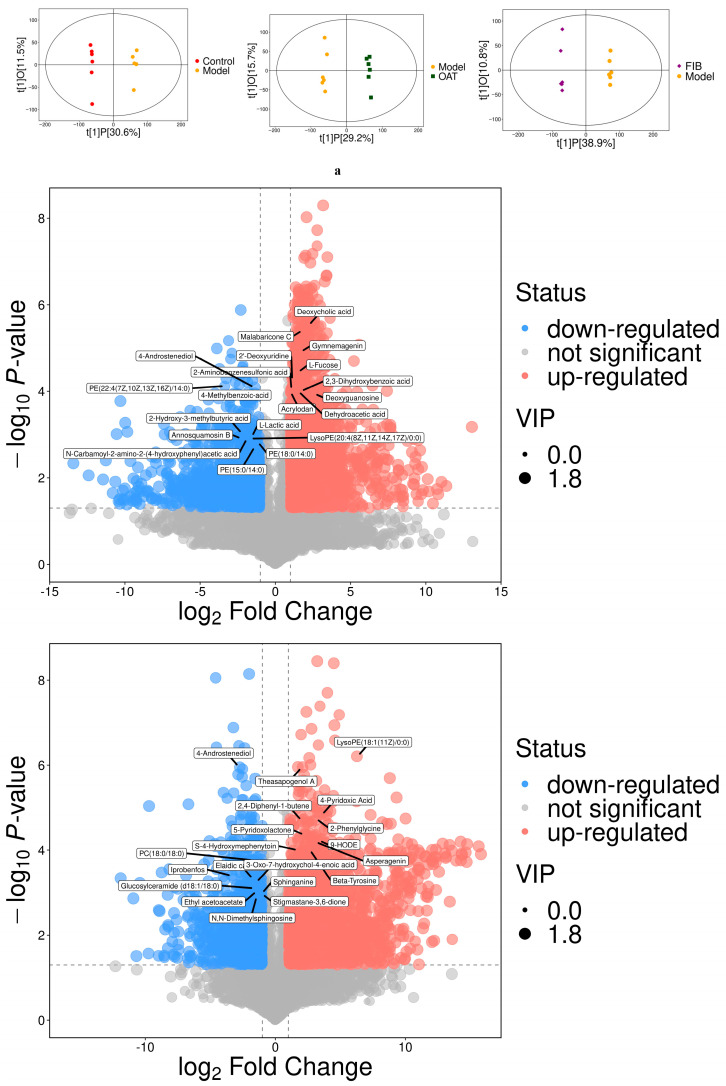

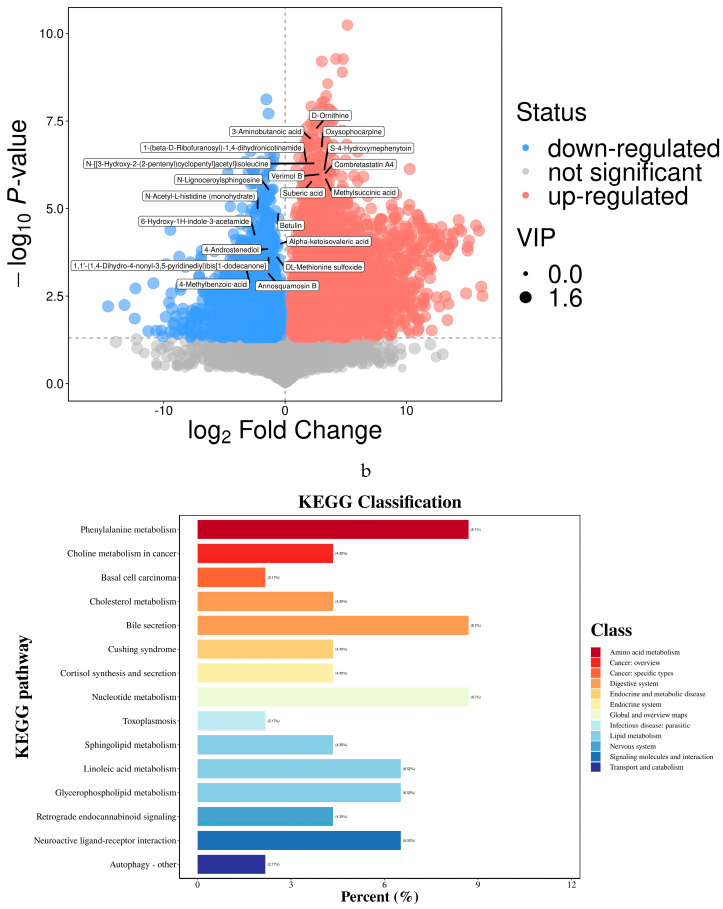

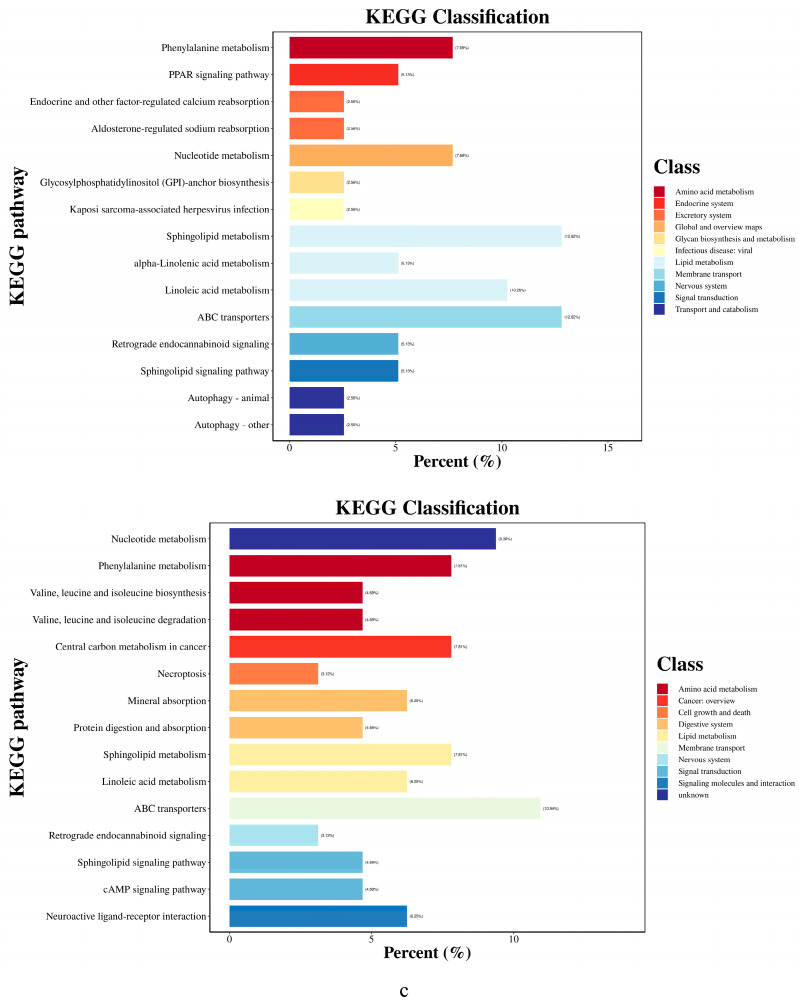
Effects of oat and oat bran on feces metabolomics analysis. (**a**) OPLS-DA of feces metabolites, (**b**) Volcano plot analysis of feces metabolites (**c**) The enrichment analysis of KEGG pathways by differential feces metabolites.

To further investigate the differences in fecal metabolite profiles between oat and oat bran interventions in IBD mice, untargeted metabolomic analysis was conducted using LC-MS. As shown in Figure 2a, the orthogonal partial least squares discriminant analysis (OPLS-DA) plot revealed clear separation between the model group and treatment groups, indicating significant alterations in fecal metabolic composition following dietary intervention. In Figure 2b, compared with the control group, the model group exhibited downregulation of several amino acids, including L-arginine, N-acetyl-L-methionine, and prolyl-arginine, while N-acetyl-L-phenylalanine was upregulated. Additionally, metabolites such as deoxyguanosine, deoxycholic acid, taurocholic acid, 2-hydroxycinnamic acid, and ursodeoxycholic acid were downregulated, whereas L-lactic acid and 2-hydroxybutyric acid were upregulated in the model group. In the oat bran-treated group, levels of 2-hydroxycinnamic acid, N-acetyl-L-phenylalanine, phytosphingosine, phenylacetylglycine, L-leucine, L-valine, and L-methionine were significantly downregulated compared to the model group. Conversely, malic acid, 3-(3-hydroxyphenyl)propanoic acid, ursodeoxycholic acid, and sphingolipids were upregulated. Similarly, in the oat-treated group, 2-hydroxycinnamic acid, phytosphingosine, and phenylethylamine were downregulated, while sphingolipids, 3-(3-hydroxyphenyl)propanoic acid, and inosine (a metabolite associated with energy metabolism) were upregulated relative to the model group. These results suggest that oat bran may primarily affect amino acid metabolism (e.g., L-leucine, L-valine), while oats may have a unique impact on neurotransmitters (such as phenylethylamine) and energy metabolism (inosine). Metabolites exhibiting significant differences were identified and annotated using the KEGG and HMDB databases, followed by pathway enrichment analysis. In Figure 2c, compared to the control group, the model group showed significant disruptions in several metabolic pathways, including phenylalanine metabolism, bile secretion, nucleotide metabolism, linoleic acid metabolism, and glycerophospholipid metabolism. In contrast, the oat intervention group primarily modulated sphingolipid metabolism, ABC transporter activity, nucleotide metabolism, linoleic acid metabolism, and phenylalanine metabolism. Similarly, the bran intervention group was associated with alterations in ABC transporters, linoleic acid metabolism, nucleotide metabolism, phenylalanine metabolism, and sphingolipid metabolism. Collectively, these findings indicate that oats and bran exert their effects mainly through the regulation of phenylalanine, sphingolipid, linoleic acid, and glycerophospholipid metabolic pathways. Notably, oat bran intervention exhibited more pronounced regulatory effects on amino acid biosynthesis as well as protein digestion and absorption, while oat intervention showed more prominent modulation of the PPAR signaling pathway and nucleotide metabolism. In summary, these findings suggest that oats and oat bran significantly alter fecal metabolite profiles in IBD mice, particularly by modulating microbial-derived metabolites involved in key metabolic pathways associated with inflammation.

### 3.3. Effect of Oats and Oat Bran on the Colonic Transcriptome

**Figure 3:**
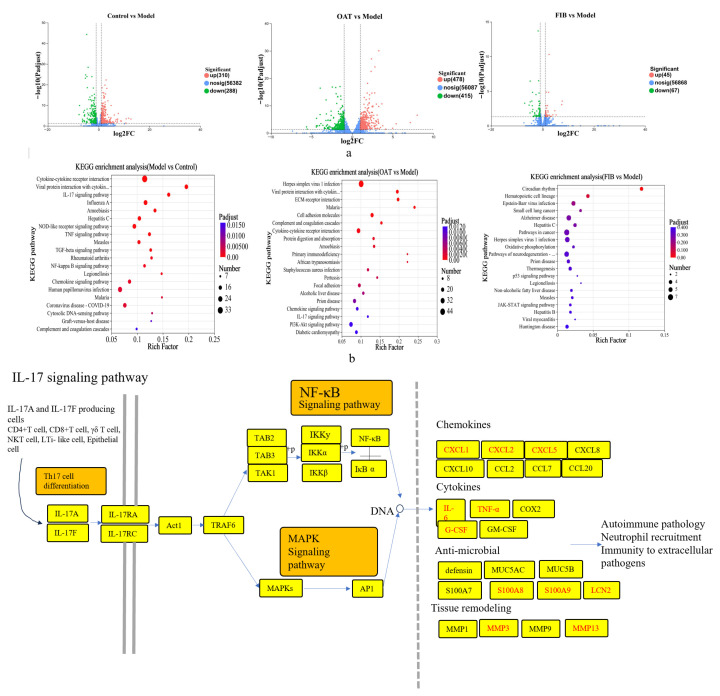

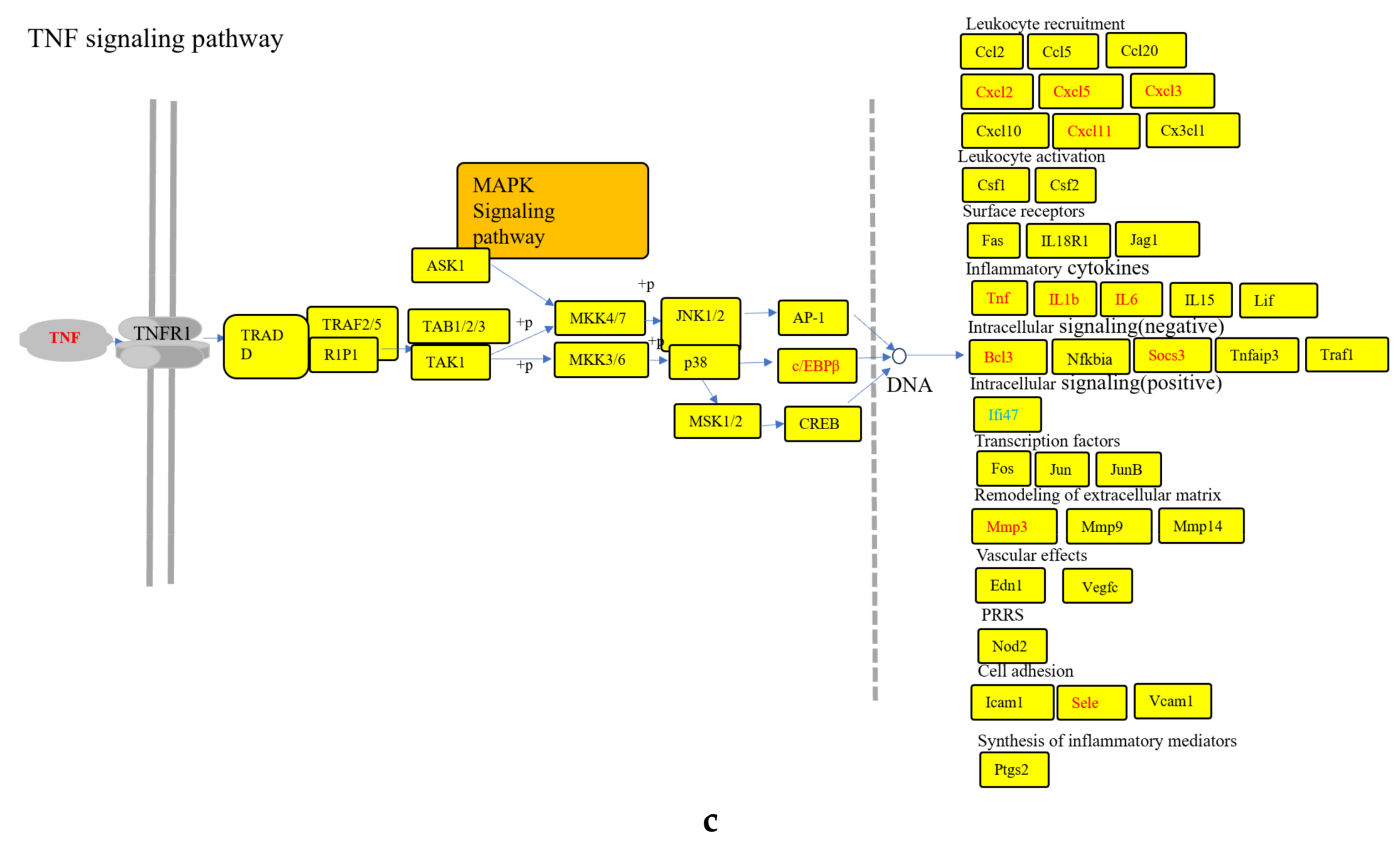
Differential gene expression analysis. (**a**) Volcano plot of the differential genes in the colon tissues of the mice. (**b**) Pathway enrichment analysis of the differentially expressed genes in the transcriptome map according to the KEGG. (**c**) The differentially expressed genes in TNF and IL-17 signaling pathways were shown and the red box represents KEGG annotated differential genes upregulated in the model group.

To further investigate the gene-level effects of oat and oat bran interventions on intestinal microbiota regulation in IBD mice, RNA-seq analysis was performed on colonic tissue samples. Differentially expressed genes (DEGs) were identified based on the criteria of |log_2_FC| ≥ 1 and *p*-value < 0.05. As shown in Figure 3a, 598 DEGs were observed in the model group compared to the control, with 310 genes upregulated and 288 downregulated. In comparison to the model group, the oat-treated group exhibited 893 DEGs (478 upregulated, 415 downregulated), whereas the oat bran-treated group showed 112 DEGs (45 upregulated, 67 downregulated). The significant difference in the number of DEGs suggests that oats induce broader transcriptional changes, likely due to their complex composition (fiber, β-glucan, and polyphenols), while oat bran’s effects are more targeted. To explore the biological significance of these changes, KEGG pathway enrichment analysis was performed for each comparison: control vs. model, model vs. OAT, and model vs. FIB groups. As shown in Figure 3b, the DEGs in the model group were significantly enriched in multiple inflammation-related pathways, including cytokine–cytokine receptor interaction, IL-17 signaling, TNF signaling, NF-κB signaling, and PI3K-Akt signaling, with IL-17 and TNF signaling pathways being the most prominently affected (Figure 3c). Notably, KEGG pathway enrichment analysis revealed that IL-17 signaling was the most consistently and strongly modulated pathway across both interventions, highlighting its key role in mediating the anti-inflammatory effects. Consistent with these enrichment results, the model group demonstrated elevated expression of numerous pro-inflammatory cytokines and chemokines, such as Cxcl1, Cxcl2, Cxcl5, IL-6, TNF-α, and G-CSF, as well as antimicrobial peptides including S100A8, S100A9, and LCN2. Dietary intervention with oats or oat bran significantly suppressed the expression of key inflammatory mediators-IL-1β, IL-6, IL-17A, TNF-α, Cxcl1, Cxcl2, Cxcl3, Cxcl5, Cemip, Ccl2, Csf3, S100A8, and S100A9 and upregulated anti-inflammatory and barrier-associated genes such as IL-10, Occludin, Claudin-1, and Claudin-5. Collectively, these transcriptomic results indicate that DSS-induced colitis causes significant alterations in gene expression linked to inflammation and barrier dysfunction, while dietary supplementation with oats or oat bran may counteract these effects by downregulating inflammatory responses and reinforcing epithelial barrier integrity.

### 3.4. Validation and Analysis of Key Genes

**Figure 4:**
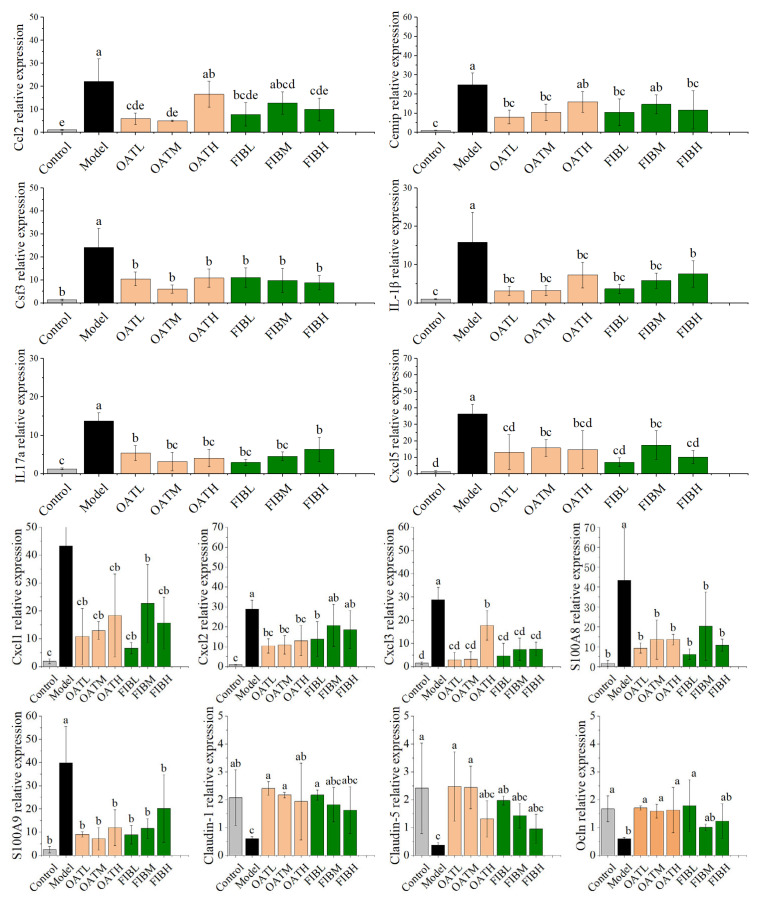
The relative mRNA expression levels of Cxcl1, Cxcl5, Cxcl2, IL-1β, cemip, S100A8, S100A9, IL-17a, Csf3, Ccl2, Cxcl3, Claudin-1, Ocln and Claudin-5 and in colon tissues was validated by RT-PCR. Different letters (a, b, c, d, e) indicate significant differences between groups (*p* < 0.05) as determined by one-way ANOVA.

To further elucidate the mechanisms by which oats and oat bran mitigate colitis and to validate transcriptomic findings, 14 related genes involved in the IL-17 and TNF signaling pathways were selected for RT-qPCR analysis. These genes were previously identified as differentially expressed in the transcriptome data following dietary intervention and are known to play critical roles in regulating intestinal inflammation. As shown in Figure 4, the mRNA expression of the pro-inflammatory cytokine IL-1β was significantly elevated in the DSS-induced model group compared to the healthy control group. However, dietary supplementation with oats or oat bran markedly reduced IL-1β expression in colonic tissues, indicating an attenuation of inflammation. Chemokines such as Cxcl1, which facilitate neutrophil recruitment and infiltration, were also significantly upregulated in the model group. RT-qPCR results further confirmed increased expression of Cxcl1, Cxcl2, and Cxcl5, key mediators of the IL-17 signaling cascade, along with the antimicrobial peptides S100A8 and S100A9. Notably, both oat and oat bran treatments significantly downregulated the expression of these inflammatory markers (Cxcl1, Cxcl2, Cxcl5, Ccl2, S100A8, and S100A9) relative to the model group, suggesting a reduction in neutrophil-driven inflammation. Furthermore, occludin, a tight junction protein essential for epithelial barrier integrity, was significantly upregulated following dietary intervention, indicating enhanced mucosal repair and reduced permeability. Overall, the RT-qPCR results were consistent with RNA-seq findings, thereby confirming the reliability of the transcriptomic data. These results suggest that oats and oat bran may alleviate DSS-induced colonic injury by suppressing key pro-inflammatory mediators-such as IL-1β and IL-17A-and by reinforcing intestinal barrier function through the upregulation of genes related to epithelial integrity.

### 3.5. Effect of Oats and Oat Bran on the Expression Level of Protein

**Figure 5:**
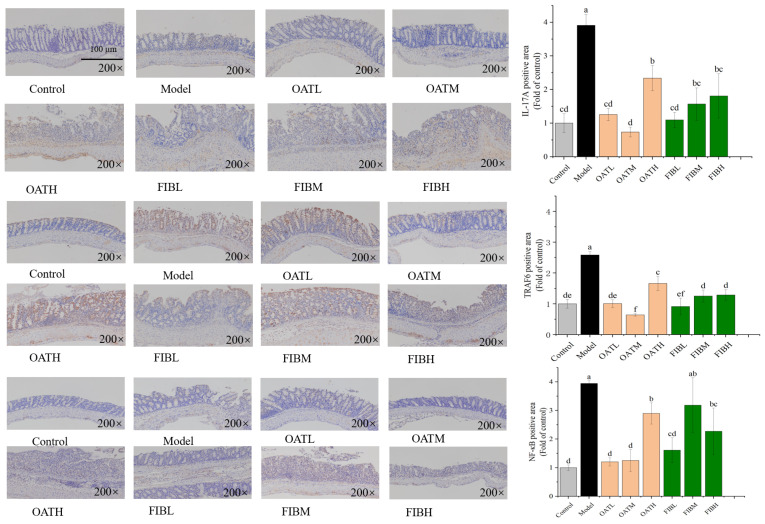
Effects of oat and bran on the intestinal barrier function in mice. The images of IL-17A, TRAF6 and NF-κB protein expression in the colonic tissues; Bar charts represent the IL-17A, TRAF6 and NF-κB positively stained area calculated by ImageJ. Different letters (a, b, c, d, e and f) indicate significant differences between groups (*p* < 0.05) as determined by one-way ANOVA.

To validate the transcriptional changes observed in the RNA-seq analyses, IHC was employed to assess the protein expression levels of IL-17A, TRAF6 and NF-κB in colonic tissues. As shown in Figure 5, the DSS-induced colitis group exhibited significantly elevated protein expression of IL-17A, TRAF6, and NF-κB compared to the control group, indicating activation of inflammatory pathways. Notably, dietary supplementation with oats or oat bran markedly suppressed the expression of IL-17A and TRAF6, with the most pronounced reductions observed in these treatment groups. Interestingly, NF-κB expression remained largely unchanged in the FIBM group, suggesting compound specific effects on this pathway. These results indicate that oats and oat bran may exert anti-inflammatory effects, at least in part, by modulating the IL-17A-TRAF6-NF-κB signaling axis. Quantitative analysis further demonstrated that DSS-induced colitis led to 3.91 fold, 2.59 fold and 3.95 fold increases in the expression levels of IL-17A, TRAF6, and NF-κB, respectively. Oat and oat bran interventions significantly attenuated these elevations, particularly at low to moderate supplementation levels. Interestingly, oats appeared more effective than oat bran in suppressing pro-inflammatory protein expression. However, higher doses of supplementation did not confer additional benefits and, in some cases, appeared to reduce efficacy or potentially exacerbate inflammation. These observations suggest that the therapeutic effects of oats and oat bran may follow a non-linear dose response relationship.

### 3.6. Molecular Docking Analysis of Key Metabolites with Target Proteins in the IL-17/NF-κB Signaling Pathway

**Figure 6:**
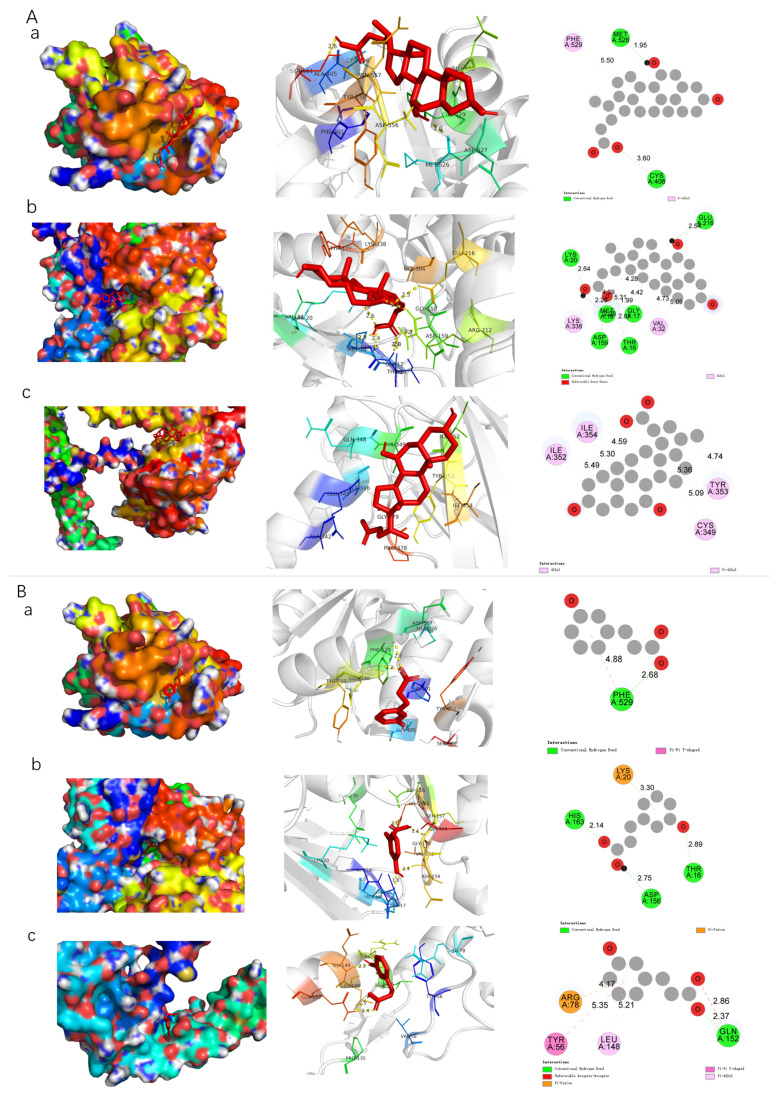

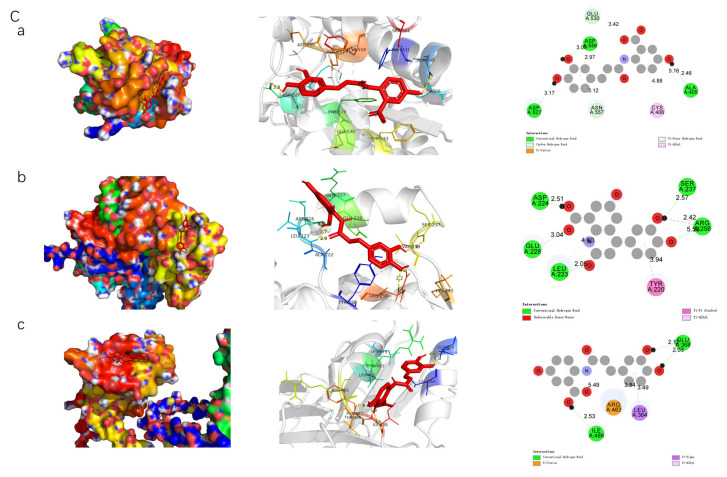
Molecular docking models. (**A**–**C**) for ursodeoxycholic acid, 3-(3-hydroxyphenyl)propionic acid and Avenanthramide C with IL-17RA, Act1 and TRAF6 protein targets. Different letters (a–c in (**A**–**C**)) represent docked IL-17RA, Act1 and TRAF6 target proteins, respectively.

To further explore the potential regulatory mechanisms of key oats and oat bran-derived metabolites on the IL-17/NF-κB signaling pathway, molecular docking analyses were conducted. Specifically, interactions between three representative metabolites, ursodeoxycholic acid, 3-(3-hydroxyphenyl)propionic acid, and avenanthramide C and three core proteins involved in this pathway (IL-17RA, Act1, and TRAF6) were evaluated based on integrated transcriptomic and metabolomic data. As shown in Table 2, three metabolites exhibited favorable binding affinities toward the selected target proteins. Molecular docking results revealed stable ligand-receptor complex formation with IL-17RA, Act1, and TRAF6, suggesting potential modulatory roles in the IL-17 signaling cascade. As shown in Table 2, both metabolites exhibited favorable binding affinities toward the selected target proteins. Molecular docking results revealed stable ligand-receptor complex formation with IL-17RA, Act1, and TRAF6, suggesting potential modulatory roles in the IL-17 signaling cascade. Among these interactions, ursodeoxycholic acid displayed a binding energy of −7.8 kcal/mol with IL-17RA, which falls within the range of medium to strong affinity (−5 to −10 kcal/mol), indicating a robust interaction. As visualized in Figure 6, ursodeoxycholic acid formed hydrogen bonds with Met526 (1.95 Å) and Cys406 (3.6 Å), as well as a P-π stacking interaction with Phe529 (5.50 Å), indicating a stable binding conformation. The binding of 3-(3-hydroxyphenyl)propionic acid to IL-17RA was also notable, with a binding energy of −5.7 kcal/mol. This compound formed multiple hydrogen bonds with Glu216 (2.54 Å), Lys20 (2.64 Å), Asp159 (2.29 Å), Thr16 (2.87 Å), Gly17 (1.99 Å), and Met18 (2.28 Å), along with π–π interactions involving Val32 (5.09 Å) and Lys338 (2.28 Å). Docking with Act1 revealed that ursodeoxycholic acid had a binding energy of −7.4 kcal/mol, indicating a strong affinity. The compound formed hydrogen bonds with several polar residues, including Glu216, Gly17, Met18, Asp159, Thr16, and Lys20, with bond lengths ranging from 1.99 Å to 4.39 Å. In the case of TRAF6, both metabolites exhibited potential binding within the protein’s catalytic domain. Ursodeoxycholic acid showed a binding energy of −7.1 kcal/mol, while 3-(3-hydroxyphenyl)propionic acid demonstrated values of −4.9 and −6.5 kcal/mol, indicating a comparatively stronger interaction for ursodeoxycholic acid. Notably, it engaged in P-π stacking and electrostatic interactions with residues Ile354, Ile352, Tyr353, and Cys349. In contrast, 3-(3-hydroxyphenyl)propionic acid formed hydrogen bonds with Gln152 and electrostatic interactions with Leu148, Tyr56, and Arg78. Avenanthramide C interacts with the protein through binding sites at Glu 368, Ile 448, Arg 483, and Leu 364. Collectively, these results demonstrate that ursodeoxycholic acid, avenanthramide C and 3-(3-hydroxyphenyl)propionic acid interact with IL-17RA, Act1, and TRAF6 through multiple bonding types, including hydrogen bonding, hydrophobic interactions, electrostatic forces, and π–π stacking. We speculate that these metabolites may directly interact with intestinal epithelial cells, potentially modulating the IL-17/NF-κB signaling pathway to exert anti-inflammatory effects and mitigate immune mediated colonic injury.

## 4. Discussion

A diet rich in whole grains, particularly those containing oats and bran-has been consistently associated with a reduced risk of chronic diseases and significant improvements in both metabolic and gastrointestinal health. Building on previous evidence that oats and oat bran can modulate gut microbiota composition and attenuate intestinal inflammation, this study demonstrates that oats and oats bran alleviate DSS-induced colitis primarily associated with mediated through modulation of the IL-17 signaling pathway and the restoration of metabolic homeostasis.

Non-targeted metabolomic analysis demonstrated that DSS induced colitis profoundly disrupted the colonic metabolic profile, with significant alterations in sphingolipids, SCFAs, bile acids, and tryptophan-derived metabolites-classes of compounds previously implicated in the pathogenesis of IBD [22]. Dietary intervention with oats and oat bran substantially mitigated these metabolic disturbances, restoring the metabolite profiles toward those observed in the healthy control group. Notably, levels of taurocholic acid and deoxycholic acid putative biomarkers of colitis were normalized following treatment, indicating a potential modulatory effect on gut microbial metabolism [23]. These metabolite changes were associated with improved disease outcomes, supporting their potential role in the protective effects.

Sphingolipids, such as phytosphingosine, are essential structural components of cell membranes and play critical roles in immune regulation. Exogenous sphingolipids have shown potential in modulating immune responses in immune mediated diseases [24]. Our study found that the content of phytosphingosine was significantly reduced following oat and oat bran supplementation, indicating that these whole grains may help alleviate mucosal inflammation by modulating sphingolipid metabolic pathways in the colon. Amino acids are also vital for maintaining intestinal homeostasis, as their metabolic functions influence various aspects of colitis progression, including symptom reduction, histological damage, inflammatory responses, apoptosis, and microbial regulation [25]. Our results revealed that phenylacetylglycine and N-(o-toluoyl)glycine were significantly upregulated, whereas arginine was downregulated in the model group, indicating substantial metabolic disturbances. Oat and bran supplementation partially reversed these changes, indicating effect on amino acid metabolism. Additionally, the increase in 3-(3-hydroxyphenyl)propionic acid highlights the potential role of polyphenol-derived microbial metabolites in the observed anti-inflammatory effects. Furthermore, previous studies have shown that arginine supplementation can reduce inflammation in DSS induced colitis models [26]. Lactate, a key metabolite in chronically inflamed tissues, was also elevated in DSS induced colitis. Excess lactate can exacerbate microbial dysbiosis and inflammatory cascades by increasing gut permeability, allowing bacterial metabolites to enter systemic circulation [27,28]. Prior research has demonstrated a positive correlation between lactic acid accumulation and IBD severity. In a clinical study of 23 patients with moderate IBD, fecal lactic acid concentrations averaged 8.9 ± 7.0 mM, significantly higher than those observed in healthy controls. This finding is consistent with the elevated lactate levels observed in the present DSS-induced colitis model [29]. Interestingly, our data also showed that oat and oat bran treatment upregulated ursodeoxycholic acid (UDCA), a secondary bile acid produced by gut bacteria. UDCA has been shown to protect colonic epithelial barrier integrity and suppress pro-inflammatory cytokine production [30,31,32]. Its upregulation may contribute to intestinal barrier restoration and attenuation of mucosal inflammation, in line with previous findings in both animal and human studies [33,34].

Transcriptomic analysis provided further insights into the molecular mechanisms potentially involved in the protective effects of oats and oat bran supplementation. The data indicated that DSS exposure significantly upregulated genes involved in pro-inflammatory signaling pathways, particularly those involving IL-17 and TNF, consistent with previous findings by Yao et al. [35]. In the DSS model, IL-17 and its downstream effectors, including IL-6 and TNF-α, were markedly upregulated, contributing to heightened inflammatory responses. In contrast, dietary intervention with oats and bran suppressed the expression of these pro-inflammatory genes while simultaneously upregulating IL-10 and regulatory T cell (Treg)-associated markers, suggesting a restoration of the Th17/Treg balance. This immunological equilibrium is a key therapeutic target in IBD, as Treg cells mitigate inflammation through the secretion of IL-10 and TGF-β [36].

We also observed significant upregulation of key inflammatory genes, such as Lcn2, Il-1β, Csf3, S100a8, S100a9, and Cxcl2, in DSS-treated mice. These genes, primarily involved in intracellular inflammatory signaling, were markedly downregulated following oat and bran supplementation. This observation is consistent with findings reported by Wu et al. [37]. Additionally, the PI3K/AKT signaling pathway, which is important for inflammation, autophagy, and maintaining the epithelial barrier, was downregulated in the oat treated group. Dysregulation of this pathway has been implicated in the pathogenesis of UC, particularly through enhanced production of pro-inflammatory cytokines and disruption of intestinal barrier integrity [38,39].

Restoring intestinal barrier integrity is crucial for mucosal healing in IBD [40]. Our results showed that oats and oat bran supplementation significantly increased the expression of tight junction proteins, particularly Claudin-5 and occludin, in colonic epithelial cells. This likely contributed to enhanced barrier function, reduced intestinal permeability, and improved immune homeostasis [36]. Notably, polyphenols and β-glucans found in oats and bran function as prebiotic substrates, modulating the gut microbiota and promoting the production of bioactive metabolites that enhance mucosal barrier integrity. These compounds may also contribute to the attenuation of low-grade inflammation and the improvement of host metabolic health.

Although both oats and oat bran exhibit anti-colitis effects, differences in their efficacy were observed. This may be due to the higher concentration of β-glucan and polyphenols (e.g., avenanthramides) in oat bran, while whole oats retain both the germ and endosperm [41]. These differences suggest that various components within whole grains, such as dietary fiber and phenolic compounds, may work synergistically or complementarily in the anti-inflammatory process [42].

The S100 family proteins S100A8 and S100A9 are well-established mediators of innate immune responses. These proteins are markedly upregulated during active inflammation, where they contribute to leukocyte recruitment and the production of pro-inflammatory cytokines. As demonstrated by Wu et al. [37]. DSS treatment induces the overexpression of S100A8/A9, activating the IL-17 signaling pathway and exacerbating colonic inflammation. Our findings are consistent with these observations, as oats and oat bran supplementation significantly downregulated S100A8/A9 expression. Moreover, active UC is also characterized by a reduction in goblet cell numbers, decreased expression of tight junction proteins, and increased intestinal permeability [40]. In this study, the upregulation of Claudin-5 in response to oats and oat bran supplementation suggests an improvement in epithelial barrier integrity and a corresponding decrease in susceptibility to chronic inflammation. This protective effect is likely attributable to the high polyphenol content present in whole oats and oat bran [43]. Together, these findings provide compelling evidence that oat and oat bran supplementation ameliorates DSS induced colitis by modulating key inflammatory pathways (IL-17, TNF, PI3K/AKT), correcting metabolic disturbances, and restoring intestinal barrier integrity. These results underscore the therapeutic potential of whole grains in the dietary management of IBD and highlight the utility of integrated multi-omics approaches in elucidating complex diet disease interactions.

## 5. Conclusions

In conclusion, this study demonstrates that dietary intervention with oats and oat bran significantly downregulates the expression of key pro-inflammatory cytokines (IL-1β, IL-6, IL-17a, TNF-α) and chemokines (Cxcl1, Cxcl2, Ccl5, Ccl2), while upregulating the expression of tight junction proteins (Claudin-1, Claudin-5, and Occludin), thereby markedly improving intestinal barrier integrity. Mechanistically, oats and oat bran may modulate the IL-17 signaling pathway by inhibiting TRAF6 and NF-κB activation, which suppresses the transcription of downstream inflammatory mediators. Moreover, molecular docking analyses further revealed that metabolites such as ursodeoxycholic acid, 3-(3-hydroxyphenyl)propionic acid, and avenanthramide C exhibited stable binding affinities to key IL-17 pathway targets (IL-17A, TRAF6, Act1) through hydrogen bonding, hydrophobic interactions, and electrostatic forces, we speculate that these metabolites may directly regulate the attenuation of colonic inflammation. Collectively, these findings elucidate the multi-level mechanisms by which oats and oat bran alleviate colitis, through modulation of the IL-17 signaling cascade and reinforcement of mucosal barrier function and provide robust theoretical and experimental support for the development of oat-based nutritional strategies in the prevention and management of IBD.

## 6. Limitations and Future Perspectives

This study explored the protective mechanisms of oats and oat bran in a murine colitis model and suggested that the IL-17 signaling pathway may play a central role in their effects. However, due to ethical constraints, human trials were not conducted, and we were unable to compare our findings with human IBD transcriptomic datasets. Future research should focus on these comparative analyses to assess the translational potential of oat-based interventions for human IBD.

While our integrated multi-omics analysis linked changes in the gut microbiota and metabolites to inflammation alleviation, the study design could not confirm whether the microbiota is an essential mediator or if specific metabolites exert protective effects. Future studies could utilize germ free animal models or antibiotic treatments to validate the role of the microbiota, and conduct targeted metabolite supplementation experiments to confirm their protective roles.

Additionally, molecular docking analysis predicted that metabolites like ursodeoxycholic acid, 3-(3-hydroxyphenyl)propionic acid, and avenanthramide C bind to key proteins (IL-17A, ACT1, TRAF6) in the IL-17 pathway. However, these results are based on computational models, not direct in vivo evidence. Further validation is needed, with a focus on understanding the cell-specific expression and subcellular localization of these proteins in the intestine to confirm the bioaccessibility of the metabolites. Immunohistochemistry and immunofluorescence techniques will be employed to analyze the expression patterns of key IL-17 signaling proteins (IL-17RA, ACT1) in colon tissue sections.

## Figures and Tables

**Table 1:** Primer sequences of genes.

Target Gene	Forward Primer Sequence	Reverse Primer Sequence
IL-1β	GAAATGCCACCTTTTGACAGTG	TGGATGCTCTCATCAGGACAG
IL-17A	TTTAACTCCCTTGGCGCAAAA	CTTTCCCTCCGCATTGACAC
Ccl2	TAAAAACCTGGATCGGAACCAAA	GCATTAGCTTCAGATTTACGGGT
Cxcl3	ACCAACCACCAGGCTACA	GAGGCAAACTTCTTGACCAT
Cxcl1	GGCTGGGATTCACCTCAA	GGCTATGACTTCGGTTTGG
Cxcl5	GGTTCCATCTCGCCATTC	GCATTCCGCTTAGCTTTC
Cemip	TCAGCCAAGGATAAACGG	TCTCGCCAACAAACAAGC
Cxcl2	CATCCAGAGCTTGAGTGTGACG	GGCTTCAGGGTCAAGGCAAACT
Ccl7	GCTGCTTTCAGCATCCAAGTG	CCAGGGACACCGACTACTG
Claudin-1	GGGTTTCATCCTGGCTTCT	GTATCTGCCCGGTGCTTT
Claudin-5	GCCTTCCTGGACCACAACA	GAGTGCTACCCGTGCCTTAA
Occludin	TGGCAAGCGATCATACCCAGAG	CTGCCTGAAGTCATCCACACTC
S100A8	TTCCTTGCGATGGTGATA	TCCTTGTGGCTGTCTTTG
S100A9	CGCAGCATAACCACCATC	TTGCCATCAGCATCATACAC
Gapdh	TGCGTGGCTTCCACACTTGCT	TTTGCCGCTCTGGGGTCTGT

**Table 2:** The results of molecular docking.

Ligands and Receptors	Binding Energy	Amino Acid Binding Site (Distance Å)		
		Hydrogen Bonding	Electrostatic Force	Hydrophobic Interaction
Ursodeoxycholic acid -IL-17RA	−7.8	Met 526 (1.95), Cys 406 (3.6)		Phe 529 (5.5)
3-(3-Hydroxyphenyl)propionic acid-IL-17RA	−5.7	Glu 216(2.54), Lys 20 (2.64), Asp 159 (2.29), Thr 16 (2.87), Gly 17 (1.99), Met 18 (4.39)		Val 32 (5.09), Ly 338s (2.28)
Avenanthramide C-IL-17RA	−7.8			Ile 354 (4.59), Tyr 353 (5.09), Ile 352 (5.49)
Ursodeoxycholic acid -Act1	−7.4	Phe 529 (2.68)		
3-(3-Hydroxyphenyl)propionic acid -Act1	−6.2	His 163(2.14), Asp 156 (2.75), Thr 16 (2.89)	Lys 20 (3.30)	
Avenanthramide C-Act1	−6.8	Gln 152 (2.37)	Arg 78 (4.17)	Tyr 56 (5.35), Leu 148 (5.21)
Ursodeoxycholic acid -TRAF6	−7.1	Asp 527 (3.17), Asp 556 (3.06), Ala 405(2.46), Asn 557(3.12), Glu 530 (3.42)		Cys 406 (4.88)
3-(3-Hydroxyphenyl)propionic acid -TRAF6	−4.9	Asp 224(2.51), Arg 256 (2.42), Ser 237 (2.57), Glu 228 (3.04), Leu 223 (2.05)		Tyr 220 (3.94)
Avenanthramide C-TRAF6	−6.5	Glu 368 (2.03), Ile 488 (2.53)	Arg 483 (3.84)	Leu 364 (3.49)

## Data Availability

The original contributions presented in the study are included in the article Further inquiries can be directed to the first author W.D. (15754367187@163.com).
